# Diagnostic Dilemma in Intra-abdominal Cancers

**DOI:** 10.31729/jnma.8919

**Published:** 2025-03-31

**Authors:** Gehanath Baral

**Affiliations:** 1 Department of Obstetrics & Gynecology, Nobel Medical College, Biratnagar, Nepal

**Keywords:** *abdominal cancers*, *cancer imaging*, *diagnostic dilemma*, *histopathology*, *tumor markers*

## Abstract

**Problem statement:**

A diagnostic dilemma exists in cancer care, especially in intraabdominal lesions. Despite the diagnostic means available so far, clinical decision-making is practically difficult due to diagnostic limitations. The inherent variation of any means influences decision-making. There is an iceberg-like diagnostic inaccuracy in revealing the specific condition or disease. This clinical decision-making gap is worrisome.

**Conclusion:**

Besides technological and bio-molecular advancements, the structured working framework would be protective and persuasive in mitigating dilemmas to some extent.

## INTRODUCTION

**Figure 1:**
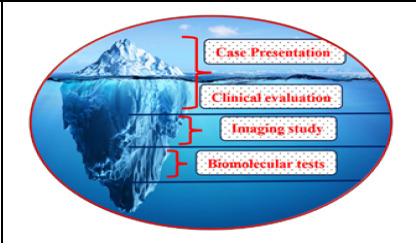
Disease Evaluation Accuracy

There are some conditions within the abdomen where the presentations of different non-cancer and cancer lesions in the abdomen are misleading.^1^ Even the organ of origin of cancer is dubious. Indirect non-invasive ways of predicting organs of origin are clinical presentations, which nowadays are undermined by trails of tests available in the vicinity; imaging features, which may suggest something; biomarkers, which share common attributes with various organs; and limited genetic markers, which are sparsely available.^2^ Each evaluation method would reveal the condition to some extent; and in combination, the diagnostic accuracy is increasingly better. Still, some conditions remain concealed or unspecified due to the lack of perfect methods or specific markers available to date or are in the developmental phase or yet to develop (Figure 1). Limitations of different imaging means and various tumor markers with variable accuracies have stressed care seekers to afford it and care providers to counsel them. Thus, the spatial differences in practice are obvious and their recommendations are also contextual. Care delivery costing is usually structured in one institution that is influenced by either business motive or service motive or mixed depending on the type of institution. This limits the implementation of applied knowledge, skills, and technology. The biological or technological pitfalls are the limiting factors in concluding results.^3^

Invasive diagnostic ways are more specific like cytology or histology but with limitations of sampling error, expertise, and ancillary imaging aid. This brings indecision and inconclusive action plans at times. Expertise includes procedure selection errors, procedural flaws, interpretation errors, and communication errors. Thus, it would be pre-analytical, analytical, and post-analytical errors.^4^ Procedure selection errors may be due to ill-defined or ill-versed pathogenesis or limitation of available means. So, these all are contextual wherever one works, he or she will have different sorts of difficulties and dilemmas. Error mitigation plans could have been incepted but at the cost of institutional strategies prevailing in place. The quality requires some reasonable investment of thought and material. However, it depends on the mission, vision, and expertise in clinical service and administrative procedures.

Visible lesions are devoid of these difficulties but those inside the body will have organ-specific limitations to draw an inference. Developmental diversity of intra-abdominal organs and tissues, their differential susceptibility pattern and shared cellular expression pattern, and ill-defined identifying characteristics lead to inaccuracy in diagnosing the disease. This condition is more pronounced in between the gastrointestinal tract and genito-reproductive organs. For instance, tumor biomarker alfa-fetoprotein may indicate origin either from the liver or germ cell tumor of the gonads; and carcinoma antigen would be from the epithelium of the bowel or ovary.^5,6^ Immunostaining may not be available at all centers and again requires the teamwork of pathologists and biomedical experts.^7^ Even metastatic deposits or lymph nodes outside the particular body part or organ would invite a dilemma if special histopathological stains are not available to distinguish the type of epithelia.^8^ Thus, its ideal solution may be a center equipped with multispecialty services everywhere which practically hardly exists nor does a perfect trail of evaluation methods exist. Situations of indecision and dilemmas will be professional challenges to the clinicians and surgeons.

Therefore, the center-specific data would differ and may not represent the population to recommend or draw a conclusion for the population. Certain tests have predictive or diagnostic value and some have prognostic significance or monitoring aid. Unknown environmental factors and ill-defined causative agents create a knowledge gap in understanding oncogenesis, thereby challenging our efforts and cost.

Some predictive models can be identified, or multidisciplinary intervention, oncogenesis research, and clinical trials are the only options to relieve our worries.^9^ Until the specific means are evolved, some kind of management model, syndromic algorithm, or working protocol can be made and piloted in the workplace. Otherwise, indecision, inaccuracy, and nonuniformity will remain. This results in inefficiency of care and a deficit in corrective measures.

## CONCLUSIONS

Service providers should always work with a window of safety in safe clinical practice especially in oncology services because of professional limitations. Working with a structured framework is safer and unidirectional by its outcome. Accuracy and efficiency of supporting means yield expected outcomes during professional discharge of duty. Judicious use of available evaluation methods and the informed margin of error are the prerequisites for reasonable care.
